# Time-course proteomics dataset monitoring HeLa cells subjected to DTT induced endoplasmic reticulum stress

**DOI:** 10.1016/j.dib.2016.07.038

**Published:** 2016-07-26

**Authors:** Zhe Cheng, Justin Rendleman, Christine Vogel

**Affiliations:** Center for Genomics and Systems Biology, New York University, New York, NY 10003, USA

**Keywords:** HeLa, Systems biology, Protein misfolding stress, ER stress, Label free mass spectrometry

## Abstract

The data described here provide an analysis of the dynamic response of HeLa cell proteome to dithiothreitol (DTT) inducing stress of the endoplasmic reticulum (ER). During ER stress, accumulation of misfolded and unfolded proteins in the lumen of the ER initiates the Unfolded Protein Response (UPR), resulting in a large-scale redistribution of proteins. We used label-free mass spectrometry to monitor the proteomic changes of HeLa cells during a 30-h time course, monitoring eight time points (0, 0.5, 1, 2, 8, 16, 24, and 30 h). The data are associated with the research article “Differential dynamics of the mammalian mRNA and protein expression response to misfolding stress” [1], which discusses a core dataset of 1237 proteins. Here, we present the extended dataset of 2131 proteins. The raw mass spectrometry data and the analysis results have been deposited to the ProteomeXchange Consortium (http://proteomecentral.proteomexchange.org) via the PRIDE partner repository with the dataset identifier PRIDE: PXD002039.

**Specifications Table**

**Table:** 

Subject area	Biology
More specific subject area	Proteomics of human HeLa cell line in response to protein misfolding stress
Type of data	Tables, figure
How data was acquired	NanoLC 2DPlus liquid chromatography system (Eksigent), LTQ Orbitrap Velos mass spectrometer (Thermo Scientific)
Data format	Raw and MaxQuant processed files (.txt)
Experimental factors	Cultured human HeLa cells treated with 2.5 mM DTT
Experimental features	Cell culture, protein extraction, protein digestion, and analysis by LC–MS/MS. Two biological replicates are provided.
Data source location	New York, NY, USA
Data accessibility	Data are within this article and are available via ProteomeXchange with identifier PRIDE: PXD002039. http://proteomecentral.proteomexchange.org/cgi/GetDataset?ID=PXD002039

**Value of the data**

• Time course monitoring eight time points for 30 h of HeLa whole cell proteome response to DTT induced protein misfolding stress (0, 0.5, 1, 2, 8, 16, 24, and 30 h). Data can be analyzed with respect to expression changes at the protein level and with respect to different phases of the stress response (time points).
• Data for more than 2100 human proteins provides substantial snapshot of proteomic response.
• Provides a high-resolution dataset of dynamic protein expression changes in mammalian cells. Protein quantitation is more accurate than traditional antibody based methods and can hence be used to disentangle protein isoforms, splice variants, or stoichiometry of complex subunits.
• Matching genome-wide mRNA data are available, collected from the same samples at the same time points and from same replicates (NCBI GEO database with the identifier GSE67901). Therefore, similarities and differences between mRNA and protein expression changes can be determined.

## 1 Data

To monitor the dynamic protein expression response in a mammalian system under stress, we designed a time-course experiment of HeLa cells being subjected to treatment with 2.5 mM dithiothreitol (DTT). We sampled the proteome at eight time points following the treatment (0, 0.5, 1, 2, 8, 16, 24, and 30 h).

## 2 Experimental design, materials and methods

### 2.1 Experimental design

**Fig. 1:**
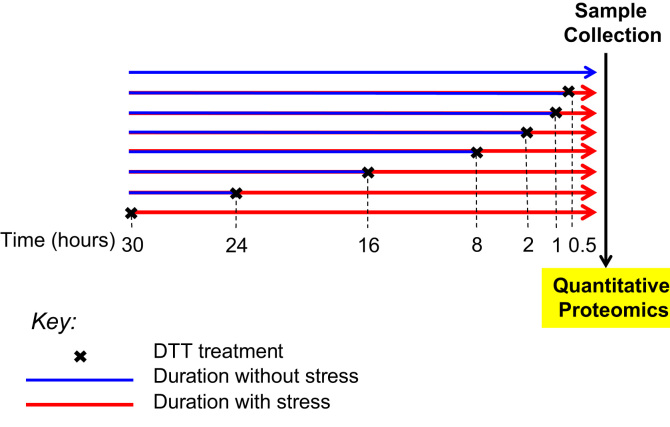
Experimental setup for the analysis of HeLa cell proteome responding to ER stress induced by DTT. Protein concentrations were measured using label-free quantitative mass spectrometry.

To account for different cellular ages at harvest, the experiment was conducted such that all cells were cultured for the same amount of time (30 h), however, with different durations of ER stress. Following this design, we grew all cells to approximately 70% confluence and started the time course when we added DTT to a 2.5 mM final concentration in the cell culture plate for the “30 h stress sample.” After six hours, DTT was added to the “24 h stress sample,” and so on for all time points. After 30 h, all samples were collected simultaneously. The treatment periods were 0, 0.5, 1, 2, 8, 16, 24, and 30 h. A label-free mass spectrometry approach was used for quantification of the proteome at each time point (Fig. 1). The experiment was conducted twice in different days to provide biological replicates.

### 2.2 HeLa cells and growth condition

HeLa cells were cultured in Dulbecco׳s Modified Eagle׳s Medium (DMEM, Sigma-Aldrich, D5796) with 10% fetal bovine serum (Atlanta Biologicals, S11150) and 1×penicillin–streptomycin solution (Corning Cellgro, 30-002-CI) at 37 °C and 5% CO_2_. When the cells were growing to approximately 70% confluency, the small molecule redox reagent DTT (Sigma-Aldrich, D9779) was added into each cell culture plate to make a 2.5 mM final concentration to induce ER stress. Eight time points were chosen to monitor the cellular stress response (0, 0.5, 1, 2, 8, 16, 24, and 30 h). DTT reduces disulfide bonds of proteins, interferes with protein folding, and leads to the accumulation of unfolded or misfolded proteins in the lumen of the ER, causing ER stress and the activation of the UPR leading to extensive proteomic changes.

### 2.3 Protein extraction, digestion and purification

After stress treatment, culture media of HeLa cells were removed, then 5 ml PBS was used to wash the cells once. The cells were digested off the plate with 0.05% Trypsin-EDTA (Gibco by Life Technologies, 25300‐054). We added 5 ml DMEM to each plate to neutralize Trypsin. Then the cells were transferred into 15 ml falcon tubes. After centrifugation at 1000 rcf for 5 min, the supernatant was removed, and cell pellets were resuspended in 400 µl Lysis buffer containing 10 mM KCl, 1.5 mM MgCl_2_, 0.5 mM DTT, and 1× protease inhibitor cocktail (cOmplete^™^, Mini, EDTA-free protease inhibitor cocktail tablets in EASYpack, Roche) in 10 mM Tris–HCl buffer (pH 8.0). The cell pellets were then Dounce-homogenized consistently among samples by 25 strokes on ice for 4 °C, and transferred to 1.5 ml Eppendorf tubes. Cell lysate was centrifuged at 1000 rcf for 5 min at 4 °C. The supernatant was saved as the cytosolic fraction, and the pellet was subjected to a single purification step using a sucrose cushion. 0.9 ml 0.88 M sucrose was carefully pipetted in to even bottom layer of 2 ml tube. Then the pellet was resuspended in 0.9 ml 0.25 M sucrose and laid carefully on top of the 0.88 M sucrose layer without any disturbance. The sucrose cushion was centrifuged at 3000 rcf for 10 min at 4 °C and the intact nuclei were located at the bottom of the tube. After washing one time with 500 µl lysis buffer, the pellet was then resuspended in 50 µl lysis buffer as nuclear fraction. The protein concentrations were determined using the Bradford protein assay (Bio-Rad, 500-0205) and the samples were diluted to 2 mg/ml concentration. For further sample preparation, 50 µl of cytosolic fraction and nuclear fraction samples were mixed with equal volume of trifluoroethanol (TFE), respectively. We added 15 mM DTT to each sample and incubated them at 55 °C for 45 min. Next, the samples were alkylated with 55 mM iodoacetamide (IAA) for 30 min at room temperature in the dark. The mass spectrometry-grade trypsin (Promega, V5073 at 1:50 v/w) was used to do the overnight digestion of the protein mixture into peptides at 37 °C. After halting the tryptic digestion by 2% formic acid (FA), the digested peptide mixtures were purified with C18 spin tips (Thermo Scientific, HyperSep, PI-89870), and then stored at −80 °C until liquid chromatography and mass spectrometry analysis.

### 2.4 Mass spectrometry analysis

Peptides were separated by reverse-phase nanoflow high-performance liquid chromatography (LC) system (Eksigent NanoLC 2DPlus), on a 15 cm Agilent ZORBAX 300 StableBond C18 reverse phase column (5065-9911, 150 mm length, 75 μm inner diameter, 3.5 μm particle, 300 Å pore size) with the gradient of 2–90% acetonitrile and 0.1% formic acid over 240 min. LC-eluted peptides were injected in-line into an LTQ Orbitrap Velos mass spectrometer (Thermo Scientific) for tandem mass spectrometry analysis. Data dependent analysis was performed in the Fourier Transform (FT) mass analyzer at a resolution of 60,000 in profile mode, and automatic gain control was set to 1E6. Dynamic exclusion was set to 90 s if mass to charge ratio (m/z) acquisition was repeated within a 45 s interval. MS2 data was collected in the Ion Trap (IT) mass analyzer in centroid mode, and the automatic gain control was set to 3E4. Injection time was 100 ms, isolation window was 2 m/z, and normalized collision energy was at 35 V. In each scan cycle, the top 20 precursor ions (MS) were subjected to fragmentation (MS/MS) via collision-induced dissociation. The experiments were conducted twice to obtain two biological replicates, and each sample was injected four times into mass spectrometer to obtain four technical replicates. The data for cytosolic and nuclear fractions and four technical replicates were combined by MaxQuant software [2] during the computational analysis, and the biological replicates were kept separately. The deposited data (PRIDE: PXD002039) contain two biological replicates (RS1 and RS3) from the label free mass spectrometry analysis of the HeLa whole cell extract.

### 2.5 Data processing

Proteome raw data files were processed and the peptide peaks were detected using the MaxQuant software (1.3.0.5) [2], then the peak lists were searched with Andromeda which is integrated in MaxQuant [3] against a database containing the translation of all predicted proteins listed in Uniprot human database (2012) and a list of commonly observed contaminants supplied by MaxQuant. The false discovery rate (FDR) for protein, peptide, and site modifications was set to 1% based on the reverse sequence of the human protein sequence FASTA file. Trypsin was used as the proteolytic enzyme while allowing up to two missed cleavages. Cysteine carbamidomethylation was selected as fixed modification, and oxidation of methionine and N-terminal acetylation were searched as variable post-translational modifications. Protein identification was performed using 20 ppm as mass tolerance at the MS level for FT mass analyzer and 0.5 Da at the MS/MS level for IT analyzer. The minimal required peptide length was set to seven amino acids. Every protein group was required to have at least one unique or razor peptide. Label-free quantitation (LFQ) with minimum ratio count set to 1 was used for quantifying the protein concentration. The complete set of parameters is provided in the MaxQuant results files deposited in ProteomeXchange (PRIDE: PXD002039). The proteingroups.txt file in the txt folder of MaxQuant result files contains the LFQ quantification information for “protein groups”. In the file, each row is named by a group of proteins, because in the mammalian complex proteomes, the peptides can be shared between homologous proteins or splice variants. MaxQuant output files with parameter settings, peptide information, and protein quantification data are provided here as Supplemental data 2.

After eliminating contaminants and reversed sequences, a total of more than 3200 proteins were quantified, of which 2828 had matched time course mRNA expression data as well. The total mRNA concentration data were deposited at the NCBI GEO database with the identifier GSE67901. When filtering by allowing one missing value for each replicate, we obtained a set of 2131 proteins with quantitative measurements of their concentrations. Supplementary Data File 3 and 4 contain the log 2 transformed normalized protein intensities (LFQ Intensities) which can be used as a proxy for protein concentration. The normalization procedure followed the default procedures as provided by the MaxQuant software (version 1.3.0.5).

Next, we prepared a core dataset of 1237 proteins which was produced by further data processing. The processing was as follows. All proteins with one or more missing values in all eight time points in either replicate were removed for generating a high-confidence dataset with 1820 proteins left. To remove the experimental variance among time points as well as keeping the general expression pattern, 95% percentile sum intensity normalization was used to normalize the protein concentration data. The top 5% most intense data in each experiment were excluded when calculating the sum intensity, which prevented extremely abundant proteins and potential outliers from dominating the normalization factor. The normalized data were then transformed by log base 2. To filter out outliers across eight time points in the data, a cutting-off procedure was applied. At first, one data point was removed at each time and the total range of variation (TRV) was calculated for the remaining data points. The TRV was calculated by subtracting the minimum from the maximum intensity data. After repeating this procedure for all eight time points, the ratio of the median TRV to the minimum TRV was calculated and used to estimate the noisiness of the data. Large TRV means that the related protein has noisy expression data. The histogram of the TRV values across all proteins was plotted to estimate the noise across all proteins. Then the threshold for tolerance level was set to 2 and proteins below the threshold were retained. A high confident dataset was generated by the strict filtering rules, although some true signals might be removed. Finally, the local regression method, locally weighted scatterplot smoothing (LOWESS) was applied to smooth the normalized and filter time course data. The LOWESS function in *R* with the default parameter settings was used. A total of 1237 proteins were left in final dataset with complete and post-processed, high-confidence protein annotations for two replicates across eight time points. This core dataset is associated with the data analyzed in publication Cheng et al. [1]. The core complete post-processed data is presented here as Supplemental data 4.

## Appendix A Supplementary material

MaxQuant output files with parameter settings and peptide and protein based information.

.

The protein concentration data, after removing genes with more than 1 missing value per replicate, followed by log base 2 transformation of the data.

.

The core protein concentration dataset, after normalization/filtering/smoothing as described in the Method.

## Footnotes

Appendix A Supplementary data associated with this article can be found in the online version at doi:10.1016/j.dib.2016.07.038.
